# Egg color variation, but not egg rejection behavior, changes in a cuckoo host breeding in the absence of brood parasitism

**DOI:** 10.1002/ece3.1096

**Published:** 2014-05-08

**Authors:** Canchao Yang, Yang Liu, Lijin Zeng, Wei Liang

**Affiliations:** 1 Ministry of Education Key Laboratory for Tropical Animal and Plant Ecology, College of Life Sciences, Hainan Normal UniversityHaikou, 571158, China; 2 State Key Laboratory of Biocontrol and School of Life Sciences, Sun Yat-sen UniversityGuangzhou, 510275, China; 3 Department of Biology, University of CaliforniaRiverside, California, 92521, USA

**Keywords:** Brood parasitism, cuckoo, egg color variation, egg rejection ability, *Leiothrix lutea*, natural selection

## Abstract

Interactions between parasitic cuckoos and their songbird hosts form a classical reciprocal “arms race,” and are an excellent model for understanding the process of coevolution. Changes in host egg coloration via the evolution of interclutch variation in egg color or intraclutch consistency in egg color are hypothesized counter adaptations that facilitate egg recognition and thus limit brood parasitism. Whether these antiparasitism strategies are maintained when the selective pressure of parasitism is relaxed remains debated. However, introduced species provide unique opportunities for testing the direction and extent of natural selection on phenotypic trait maintenance and variation. Here, we investigated egg rejection behavior and egg color polymorphism in the red-billed leiothrix (*Leiothrix lutea*), a common cuckoo (*Cuculus canorus*) host, in a population introduced to Hawaii 100 years ago (breeding without cuckoos) and a native population in China (breeding with cuckoos). We found that egg rejection ability was equally strong in both the native and the introduced populations, but levels of interclutch variation and intraclutch consistency in egg color in the native population were higher than in the introduced population. This suggests that egg rejection behavior in hosts can be maintained in the absence of brood parasitism and that egg appearance is maintained by natural selection as a counter adaptation to brood parasitism. This study provides rare evidence that host antiparasitism strategies can change under parasite-relaxed conditions and reduced selection pressure.

## Introduction

Avian brood parasites lay their eggs in other birds' nests and, thus, transfer the cost of parental care to their hosts. This parasitic behavior is a selection pressure that drives the evolution of host defences against brood parasitism (Davies 2000). Cuckoos are well-studied Old World brood parasites that exploit a wide range of songbirds as hosts, and to decrease the risk of brood parasitism, songbird hosts have evolved a variety of defence strategies that in turn select for corresponding counter adaptations within cuckoos. These reciprocal evolutionary adaptations constitute an “arms race” and provide an exceptionally good model system for understanding the process of coevolution processes (Rothstein 1990).

Several defensive strategies against cuckoo parasitism have been documented in host songbirds. For example, the ability to discriminate hosts' own eggs and reject foreign eggs is a pervasive defensive behavior to cuckoo parasitism apparent in many cuckoo hosts (Brooke and Davies 1988; Davies and Brooke 1989a). Another strategy is egg color polymorphism that allows hosts to visibly differentiate their eggs from cuckoo eggs via increased variation in egg appearance between clutches (Swynnerton 1918; Jackson 1998) or reduced variation in egg appearance within clutches (Davies and Brooke 1989b; Jackson 1998). As both egg polymorphism and egg discriminative ability protect against cuckoo parasitism, they should be maintained by natural selection in the presence of cuckoo parasitism and decay in the absence of this selection pressure. Determining whether the presence and absence of cuckoo parasitism is a key driver for functional behavioral traits under reciprocal selection is pivotal to understanding the dynamics of a coevolutionary arms races. However, rigorous empirical or experimental evidence from the field remains scant in the wild (but see Lahti 2005, 2006; Kuehn et al. 2014).

An effective method to quantify the intensity and duration of natural selection is to measure the changes in biological traits that occur when the selection pressure is released (or removed. Evolutionary changes are particularly likely in cases when traits are organized into functional suites (Lahti et al. 2009), and removal of an agent of selection is supposed to lead to rapid evolutionary responses (Lahti 2005). Human-driven species introductions present a rare experimental opportunity to test these predictions by creating natural variation in selective pressures over space and time. As the evolution of populations of introduced species can be very rapid (even for vertebrates), comparisons between introduced and native populations can provide insight into the effects of natural selection on wild populations (Berry 1964). For example, egg rejection behavior in village weaverbirds (*Ploceus cucullatus*) in the introduced populations in Hispaniola and Mauritius was compromised by changes in egg appearance, but there was no significant decline in their ability to recognize foreign eggs comparing those of a native African population (Lahti 2006), because their egg recognition system remained intact despite the decay of egg coloration distinctiveness and consistency over evolutionary time (Lahti 2005, 2006).

In this study, we compare introduced and native populations of a cuckoo host system to investigate egg rejection behavior and egg color polymorphism. Specifically, we examined two populations of red-billed leiothrix (*Leiothrix lutea*), a common cuckoo (*Cuculus canorus*) host: a population introduced to Hawaii 100 years ago where cuckoos are absent and in a population in its native range in China where cuckoos are present. To test the hypothesis that increased interclutch variation and intraclutch consistency are counter adaptations to brood parasitism, we used a tetrahedral color space model incorporating information about the hosts' visual system to compare egg color variation in the two populations. Overall, we aimed to determine the extent that variation in egg recognition and egg color is maintained when a former, strong selection pressure is absent.

## Materials and Methods

### Study species and study areas

**Figure 1:**
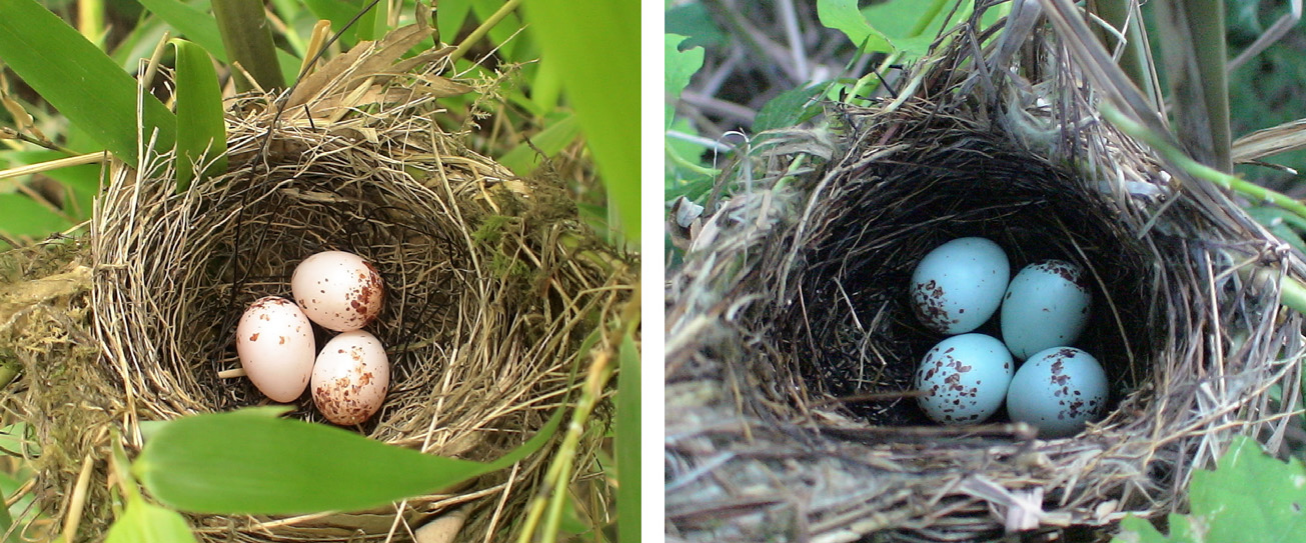
Eggs and nests of the red-billed leiothrix (*Leiothrix lutea*). Photograph by C. Yang.

The red-billed leiothrix (Leiothrichidae) is a medium-sized, green-and-yellow babbler with a conspicuous bright red bill (del Hoyo et al. 2007). It is a common resident species native to the Himalayas, Myanmar, southern China, and Vietnam where it is found in a wide variety of habitats, including broadleaf evergreen, pine and mixed forests, forest edges, secondary growth, and varied scrub, from sea level to 4000 m a.s.l. (Male et al. 1998). It was recorded as one of the normal hosts of the common cuckoo in India (Baker 1942) and builds open-cup nests in branches in dense vegetation and lays polymorphic eggs that vary in color from white to bluish-green with blood-red marking patterns (Fig. 1). Its conspicuous features, melodious song, and extremely active habits have made it a popular cagebird (Long and Tingay 1981). The red-billed leiothrix has been introduced to several countries, and feral populations exist in Japan, France, Italy, Hong Kong, and throughout the Hawaiian archipelago (del Hoyo et al. 2007). The red-billed leiothrix is a known regular host of the common cuckoo in India, and cuckoo eggs recorded in their nests are immaculate blue (Baker 1942). Alhough brood parasitism by cuckoos has not been reported in populations of red-billed leiothrix in southern China, this species is confronted with parasitic pressure from multiple sympatric cuckoo species (Yang et al. 2012a,b).

From April to August 2012, we studied a native population of red-billed leiothrix at Kuankuoshui Nature Reserve (28°10′N, 107°10′E) in Guizhou Province, south-central China. The habitat is subtropical broadleaved and mixed forest at an altitude of about 1500 m with an annual average temperature of 13.6°C and an average precipitation of 1210 mm (also see Yang et al. 2013). Within this region, several sympatric cuckoo species can be found, including the common cuckoo (Yang et al. 2012a,b2012b).

From April to May 2012, we studied an introduced population of red-billed leiothrix at the Kipuka Puaulu forest (19°26′N, 155°18′W) in Hawaii Volcanoes National Park on the Island of Hawaii, USA. This species was first introduced to the Hawaiian archipelago in 1911 (Fisher and Baldwin 1947), intentional releases to the wild continued after 1918 (Caum 1933), and it has since become widespread and common in both native and exotic wet forests on all of the islands (Male et al. 1998). The study site, Kipuka Puaulu, is located at 1,200 m a.s.l. on the southeastern flank of Mauna Loa volcano and comprises 42 ha of virgin, native Hawaiian forest surrounded by lava rock fields with scattered shrubs and trees. The site has an annual average temperature of 16°C and an average precipitation of 1500 mm (Mueller-Dombois & Lamoureux 1967).

### Artificial parasitism experiments

To investigate the egg recognition in leiothrix, we conducted artificial parasitism experiments in both the native and introduced populations. We used nonmimetic, pure white, model eggs made of polymer clay to parasitize leiothrix nests. Nests were found by systematically searching the leiothrix habitats. Model eggs were inserted into the host nests during their early incubation period, and experimental nests (*n* = 18) were then monitored on a daily basis for 6 days. Host responses to the model eggs were classified as acceptance (i.e., the model eggs were warm and being incubated) or rejection (i.e., the model eggs were pecked or had disappeared). Any nests that parents had deserted were excluded from analyses. A control experiment (involving a nest visit with no manipulation of eggs) was performed in the native Chinese population and found no rejection or desertion occurred (*n* = 18). The Fisher's exact test was used to compare the rejection frequencies between the native and the introduced population. Data analyses were performed in SPSS 13.0 for Windows (SPSS Inc., Chicago, IL).

### Intra- and interclutch variation in egg color

A spectrophotometer (Avantes-2048, Apeldoorn, the Netherlands) was used to quantify egg color in 23 nests in each population, respectively. Six values for egg reflectance were measured for each egg: three reflectance values of egg background color (averaged for each egg) and three reflectance values of the egg markings (average for each egg). All the measurements of background color and markings obtained on all the eggs in a nest were averaged to arrive at a single value per clutch. Reflectance spectra were processed through tetrahedral color space (Goldsmith 1990) using the tetracolorspace program (Stoddard and Prum 2008). Spectral sensitivity curves for leiothrix retinas were obtained from Maier (1992). The distribution of egg colors was mapped onto a unit sphere centered on the achromatic origin using a Robinson projection, which includes horizontal (RGB) and vertical (UV) components of hue. We did not include egg markings as a trait because leiothrix recognizes their eggs using egg color rather than markings (C. Yang, Y. Liu, and W. Liang, unpubl. data).

To compare intraclutch variation in egg color between the native (*n* = 23 nests of the whole clutch) and the introduced populations (*n* = 23 nests), we calculated the standard deviation of hue (including RGB and UV), chroma, and brilliance of each clutch (Yang and Liang 2013) and compared these between native and introduced populations using independent samples t-tests or Mann–Whitney *U*-tests. To compare interclutch variation in egg color, we calculated the RGB component of hue, UV component of hue, chroma and brilliance of each clutch and compared these between the native and introduced populations using Levene's Test for Equality of Variances. Statistical analyses were performed using IBM SPSS 20.0 (IBM Inc., Armonk, NY) and data presented as mean ± SE. Spectral analyses were performed in MATLAB 7 for Windows (MathWorks, Inc., Natick, MA).

## Results

### Egg rejection behavior

Results of the artificial parasitism experiment revealed that there was no statistical difference in egg rejection rates were identical between birds in the native and the introduced populations, except for two cases of desertion that were not counted (Table 1; *χ*^2^ < 0.001, df = 1, *P* = 1.00, Fisher's exact test). The majority of rejections occurred in the first day of the experiment, both in the native population (89%, 17/19) and the introduced population (94.1%, 16/17), with no difference between the two populations (*χ*^2^ = 0.25, df = 1, *P* = 1.00, Fisher's exact test).

**Table 1:** Results from artificial parasitism of the red-billed leiothrix (*Leiothrix lutea*). Numbers in the parentheses are nests where egg rejection occurred on the first day of the experiment

	Rejected	Accepted	Deserted	Total
Native population (KKS, China)	19 (17)	0	1	20
Introduced population (Hawaii, USA)	17 (16)	0	1	18

### Intra- and interclutch variation in egg color

**Figure 2:**
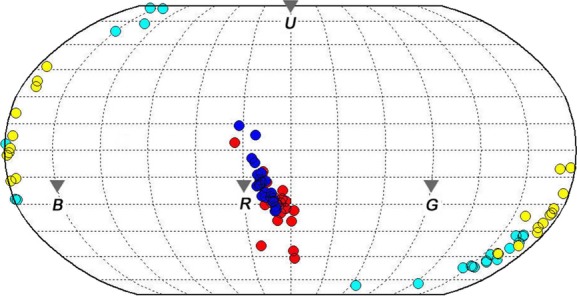
Robinson projection of egg color hue of leiothrix in China (*n* = 23 nests) and the Island of Hawaii, USA (*n* = 23 nests). Pale and dark blue circles refer to the background and marking color of eggs in the native population, respectively. Yellow and red circles refer to the background and marking color of eggs in the introduced population, respectively. Gray triangles indicate projections of the blue (B), green (G), and red (R) (i.e., the short (s), medium (m), and long (l) wavelength) vertices of the tetrahedron.

**Figure 3:**
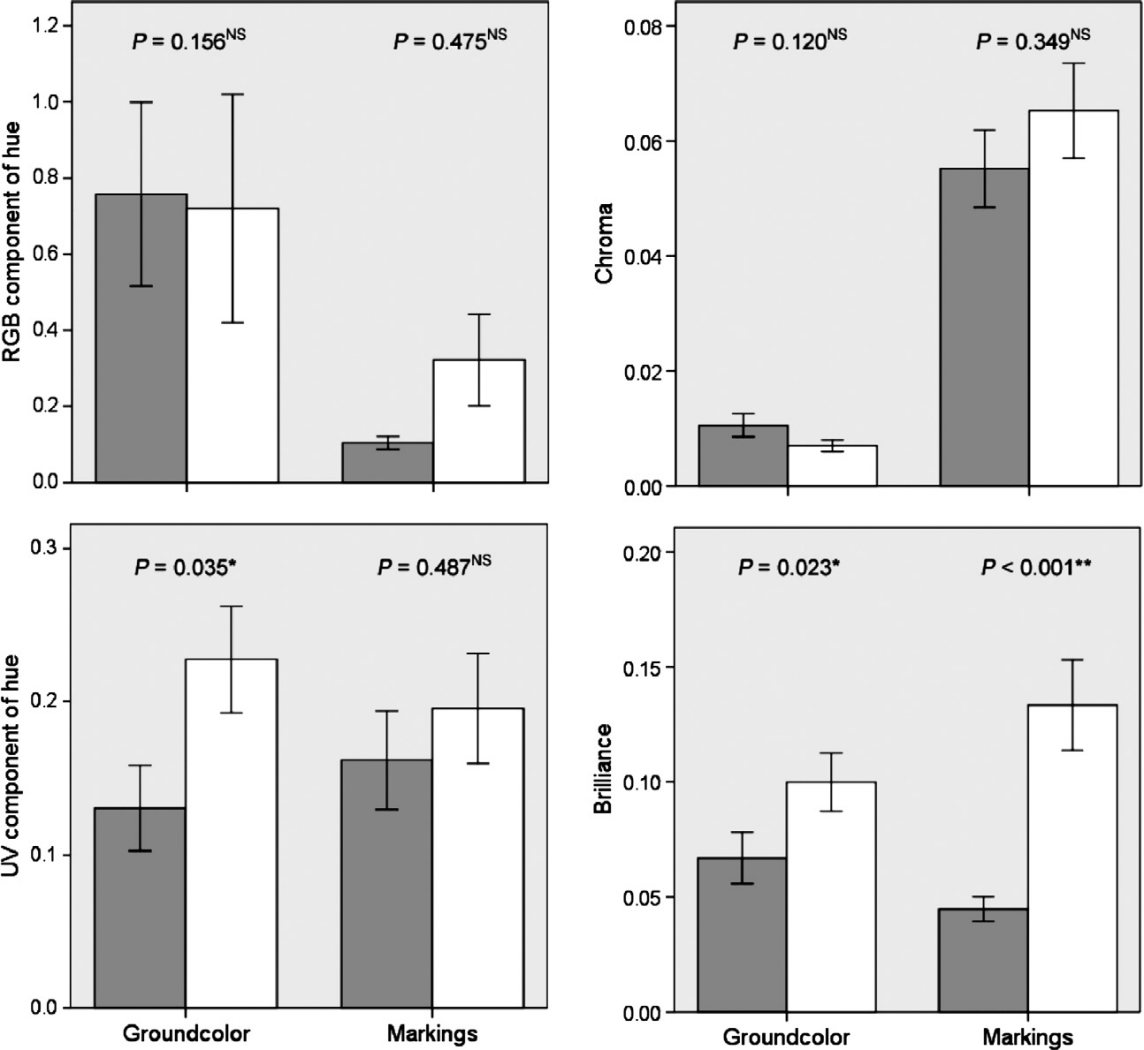
Comparison of intraclutch variation of leiothrix egg color between the native (China) and introduced (Island of Hawaii, USA) populations (mean ± SE) using either an independent samples t-test or Mann–Whitney *U*-test. Gray and white bars refer to native and introduced populations, respectively. *P* > 0.05^NS^; *P* < 0.05*; *P* < 0.01**.

**Figure 4:**
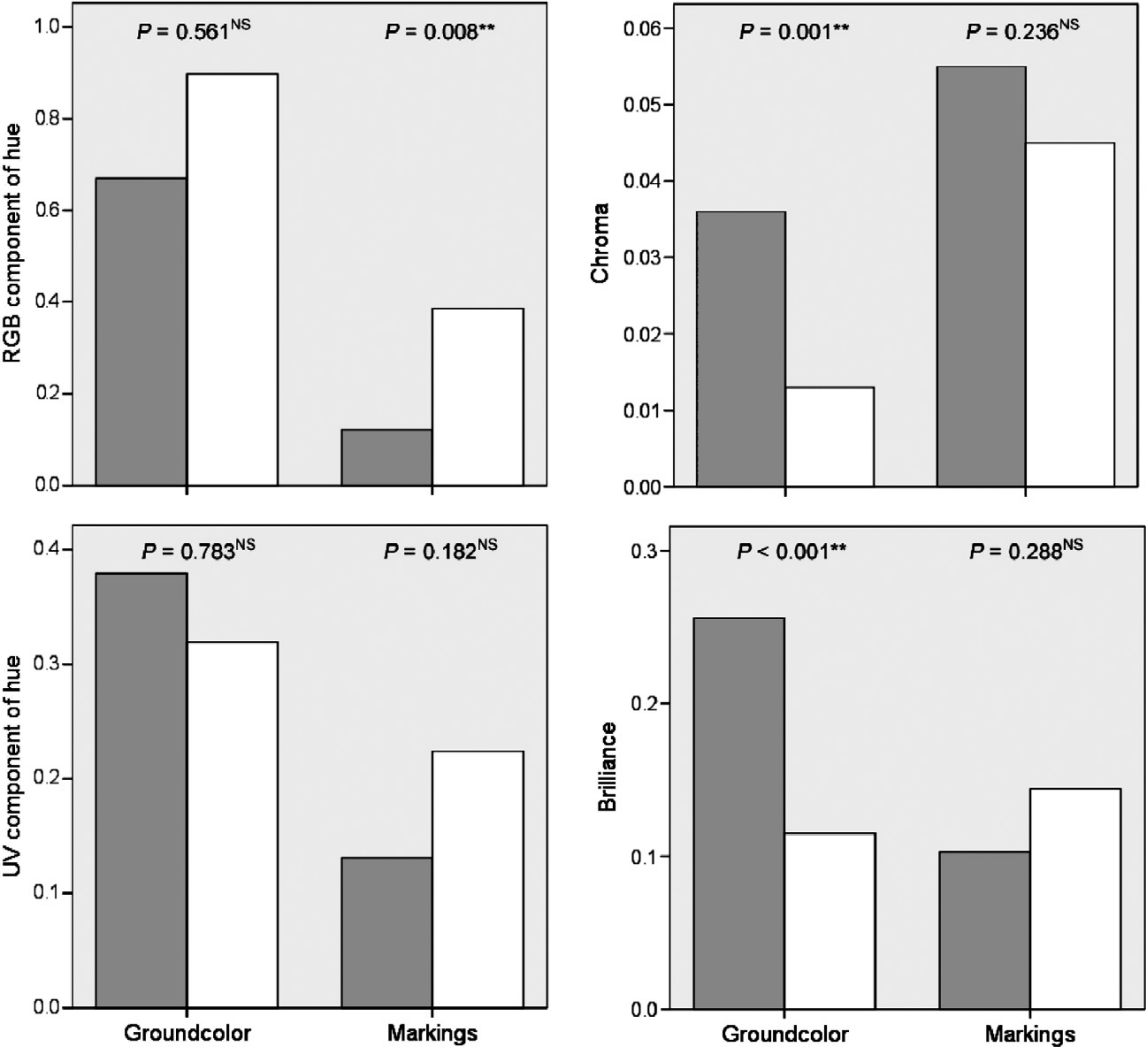
Comparison of interclutch variation of leiothrix egg color between native (China) and introduced (Island of Hawaii, USA) populations using Levene's Test for Equality of Variances. Presented values are standard variation, and gray and white bars refer to native and introduced populations, respectively. *P* > 0.05^NS^; *P* < 0.01**.

Robinson projection provided a preliminary result for egg color variation in the two leiothrix populations (Fig. 2). For intraclutch variation, differences were detected for the UV component of ground-color hue (*t* = −2.17, df = 44, *P* = 0.035, independent samples *t*-test) and ground-color brilliance (*Z* = −2.27, *P* = 0.023, Mann–Whitney *U*-test) and markings (*Z* = −3.66, *P* < 0.001, Mann–Whitney *U*-test). No intraclutch differences were found for other color parameters for the native and introduced populations (RGB component of ground-color hue: *Z* = −1.42, *P* = 0.156; RGB component of hue of markings: *Z* = −0.71, *P* = 0.475, Mann–Whitney *U*-test; UV component of hue of markings: *t* = −0.70, df = 44, *P* = 0.487; chroma of ground-color and markings: *t* = 1.59, df = 44, *P* = 0.120 and *t* = −0.95, df = 44, *P* = 0.349, respectively, independent *t*-test). All differences in egg color between the two populations exhibited the same pattern whereby intraclutch variation in the native population was smaller than for the introduced population (Fig. 3). There was greater interclutch variation in the chroma (*F*_1,44_ = 12.40, *P* = 0.001) and brilliance (*F*_1,44_ = 17.18, *P* < 0.001, Levene's Test for Equality of Variances; Fig. 4) of ground-color in the native population compared with the introduced population but not for other color parameters (RGB component of hue of ground-color: *F*_1,44_ = 0.34, *P* = 0.561; UV component of ground-color hue and markings: *F*_1,44_ = 0.08, *P* = 0.783 and *F*_1,44_ = 1.84, *P* = 0.182, respectively; chroma of markings: *F*_1,44_ = 1.44, *P* = 0.236; brilliance of markings: *F*_1,44_ = 1.15, *P* = 0.288), except for the RGB component of hue that followed an opposite pattern (*F*_1,44_ = 7.68, *P* = 0.008; Fig. 4).

## Discussion

Egg rejection ability and egg color variation in songbirds species are considered to be defensive strategies that evolved to limit cuckoo parasitism and, hence, are traits believed to be under strong selection. Here, we have shown that internest variation in the chroma and brilliance of egg background color and intranest variation in the UV component of hue and the brilliance of ground-color and markings are lower in populations of red-billed leiothrix when cuckoos are present. To our knowledge, this is the one of the first examples that host antiparasitism strategies of hosts can change under a parasite-relaxed conditions and reduced selection pressure.

### The maintenance of egg rejection ability is independent of cuckoos

Our results indicated no difference in egg recognition or rejection ability between the introduced and native populations of leiothrix because almost all foreign eggs were rejected on the first day of the experiment by both groups. Similar results were found by Lahti (2006) in an experiment conducted on two introduced populations of village weaverbird. These results indicate that hosts' egg recognition ability in hosts decays very slowly and can be maintained for a long time following a species' escape from brood parasitism. Rothstein (2001) also found that host populations descended from lineages likely to have been parasitized, but not parasitized currently, continue to eject nearly 100% of nonmimetic eggs. The persistence of this adaptive behavior under relaxed selection pressure may indicate that it is not costly to maintain (Kuehn et al. 2014). In line with host-parasite systems, previous studies showed that antipredation behavior requires several hundreds to even thousands of years to disappear in island populations after a relaxation in predation pressure (Magurran et al. 1992; Blumstein et al. 2000).

However, the acquisition of egg recognition can occur quite rapidly. For example, after their initial exposure to parasitism by cuckoos, the egg rejection rates in azure-winged magpies (*Cyanopica cyana*) increased from 0% to 30–60% in just 20 years (Nakamura 1990; Nakamura et al. 1998). Similarly, the egg rejection rate of the magpies (*Pica pica*) to eggs of the great spotted cuckoo (*Clamator glandarius*) increased from 0% to 10% in just 10 years (Soler et al. 1994). This rate of behavioral change is too rapid to be a consequence of genetic change (Davies and Welbergen 2009) and is more likely to be the result of transmission via social learning (Soler 2011); a suggestion that was confirmed by the mobbing behavior of hosts (Davies and Welbergen 2009). The acquisition of egg recognition may occur more rapidly than its loss because it occurs under strong, directional selection pressure on the particular hosts. In the absence of brood parasites, however, selection pressure is not reversed but simply released, such that the trait continues to evolve under neutral selection and only a new selective pressure can accelerate its rate of change in that particular trait. For example, strong egg rejection rates in hosts result in rejection errors, and subsequently, rejection of their own eggs and natural selection will favor reduced egg rejection behavior in hosts. While social learning may influence the uptake of egg rejection behavior, our finding of persistent egg rejection in populations free from brood parasites supports the idea that egg recognition behavior in leiothrix has a strong genetic basis.

### Differences in egg color between native and introduced host populations could be the result of relaxed selection of cuckoo parasitism

We found increased interclutch variation and intraclutch consistency, but no change in the intraclutch consistency of egg background color and markings in the native population compared with the introduced populations. However, the RGB component of the hue disparity of egg markings between nests followed the opposite pattern, possibly because egg markings are not a critical cue in egg recognition. For example, a field experiment revealed that leiothrix uses egg ground-color rather than markings to recognize their own eggs and discriminate alien eggs (C. Yang, Y. Liu, and W. Liang, unpubl. data).

It has been proposed that random mechanisms such as gradual genetic drift and founder effects can result in phenotypic shifts in recently colonized island populations (Lande 1976). However, it is unlikely that such random processes alone caused the observed differences in eggs detected here (Fig. 3 and 4) because (1) current populations across the Hawaiian archipelago were introduced multiple times between 1911 and 1937 (Fisher and Baldwin 1947), and the overall population size here is estimated to be tens of thousands in Hawaii (Male et al. 1998) and regarded stable (Ralph et al. 1998); and (2) founder effects could not be the only process driving changes in egg color in the introduced population. If founder effects played a deterministic role regarding differences in egg color between introduced and native populations, the color spectrum was more disperse in the studied population than which in native populations, whereas the effect of founder effect would be more dispersed in the native population. Our results do not support this and we instead posit that release from cuckoo parasitism-driven selection pressure plays a role in driving shifts in egg color.

Hypotheses explaining intra- and interclutch variation in egg color polymorphism have spawned a cottage industry of empirical testing; however, the experimental evidence supporting this hypothesis has been mixed (Stokke et al. 1999, 2002; Karcza et al. 2003; Avilés et al. 2004). For example, Yang et al. (2010) revealed that brood parasitism from the common cuckoo exerts a disruptive selection on its host, the ashy-throated parrotbill (*Paradoxornis alphonsianus*), driving coevolution of polymorphic egg color in these two species. Empirical studies comparing host rejection behavior and egg color variation between native and introduced populations, with and without cuckoo parasitism, have rarely been reported before. One impressive and systematic study from Lahti (2005) showed that both the intraclutch consistency and interclutch variation in egg appearance of the village weaverbird decreased in two introduced populations which had existed without egg-mimicking brood parasites for more than 100 and 200 years, in comparison with the native population. Interestingly, in that study, the difference in interclutch variation between the source and the introduced populations was only significant when comparing the older introduced population (i.e., 200 years), although a trend in the general direction was observed in the younger introduced population (Lahti 2005). Here, we report that significant changes in interclutch variation and intraclutch consistency in egg color between native and introduced populations can occur on a timescale of approximately <100 years. These results further support the hypothesis that polymorphic egg appearance is a strongly selected trait under brood parasitism pressure of brood parasitism.

In comparison with egg rejection behavior, changes in egg color variation apparently evolve rapidly (e.g., in approximately 100 years in the present study). This disparity may be a consequence of the fact that egg appearance is also under concurrent selection to optimize factors other than minimizing brood parasitism, such as maximizing solar radiation and minimizing predation (Moreno and Osorno 2003). Egg coloration is likely to be a consequence of local adaptation to specific environments (Mathys and Lockwood 2011) and may be correlated with female's genetic quality (Moreno and Osorno 2003), which interact with each other when multiple sources of selection (natural and sexual) coexist. Thus, geographic differences between native and introduced populations are likely to have a far greater influence on egg appearance than on egg rejection behavior. As the investment of eggshell pigments is costly (Moreno and Osorno 2003; López-Rull et al. 2008; Sanz and García-Navas 2009), subsequent selection after removal of brood parasitism may favor hosts to reduce this unnecessary expenditures, resulting in decreased population variation in egg coloration. However, it remains unclear to what extent biotic and abiotic constraints shape egg color variation and egg rejection behavior. Combined, we provide rare empirical evidence that egg traits functioned as cues for recognizing alien eggs by hosts changed due to relaxed selection, as opposed to egg rejection ability *per se*. Since both egg coloration and recognition have a heritable basis and are probably driven by both natural and sexual selection (Moreno and Osorno 2003), an investigation into the genetic architecture of complex traits and their changes under context-dependent selection and other evolutionary processes is needed.
